# Development and Validation of the Digital Life Balance (DLB) Scale: A Brand-New Measure for Both Harmonic and Disharmonic Use of ICTs

**DOI:** 10.3390/bs12120489

**Published:** 2022-12-01

**Authors:** Mirko Duradoni, Elena Serritella, Claudia Avolio, Claudio Arnetoli, Andrea Guazzini

**Affiliations:** Department of Education, Literatures, Intercultural Studies, Languages and Psychology, University of Florence, 50135 Florence, Italy

**Keywords:** digital life balance, psychology of harmony, well-being, behavioural addiction, problematic ICT use

## Abstract

The use of new technologies and information communication technology services (ICTs) has greatly increased, especially after the COVID-19 pandemic, resulting in an irrevocable change in people’s work-life balance (WLB). Despite the thriving literature on the dysfunctional use of new technologies, a functional use of ICTs also seems to be possible. Inspired by the theory of psychology of harmony and referring to behavioral addiction models and substance use models, we defined the construct of digital life balance to indicate a harmonic balance between digital life and real life. In this context, the imbalance between online and offline life may reflect a dysfunctional use of ICTs and can be seen as a process of disharmonization. With this perspective in mind, the aim of this study was to develop a dedicated measuring instrument that could capture both people’s balanced and unbalanced use of ICTs. Through two cross-sectional studies (Study 1= 1473 participants; Study 2 = 953 participants), we validated the scale internally and externally. In line with the literature, Digital Life Balance scores appeared to be negatively associated with addiction measures and positively associated with well-being measures. In conclusion, the Digital Life Balance (DLB) Scale appears to be a reliable (ω = 0.89) and valid instrument to investigate people’s harmonic and disharmonic use of ICTs.

## 1. Introduction

Since the spread of the COVID-19 virus in early 2020, the social, labor and economic balances of people’s lives have dramatically changed [1,2,3]. For some workers, social distancing and lockdown restrictions made it difficult to physically go to the office and interact with co-workers and clients [1]; for others, the work stoppage meant layoffs or salary suspensions [2]. Family life was also affected by the pandemic; think of parents who had to manage “online distance learning” (ODL) while continuing to work [3,4] or those who had to learn to work from home [1]. The latter is probably the most observable effect of the COVID-19 pandemic on the work environment. The concept of work from home (WFH) has not only revolutionized the way that individuals work (e.g., they have had to change their routines and learn to use special software and equipment that they may not have used before), but it has also radically changed their non-work life balance, both in terms of space (e.g., the need to carve out a work space in their own home and manage the presence of other members of the household who also need to stay home) and time (e.g., balancing demands of and responsibilities towards family with those of work). As a result of this change, their work-life balance (WLB) has also been altered, perhaps, irrevocably [5]. 

An interesting way of shedding light on such dynamics is to use the principles of the Psychology of Harmony [6]. The term “harmony” in Western culture originated from Pythagoras and the harmonic progression in music. For Western philosophers, harmony was seen as a pre-given order to be maintained [6]. For the Eastern world, the concept of harmony draws its origin from the notion of He: it is a status (not a pre-given order) with the goal of living in harmony with nature and other human beings [6,7]. Despite these differences, both cultures see harmony as a dynamic process (not a state) which is “generated through harmonization and based on balancing different elements into an organic whole” [6] (p. 3). Moreover, although the terms balance and harmony are often used interchangeably, balance is actually only one element of the broader notion of harmony [8]. 

Harmony can be disrupted in various areas: within the individual, between individuals, between human beings, and within the natural world/universe [6,7]. The most interesting aspect from the perspective of this research is the harmony in various spheres of individuals’ lives, which was greatly impacted by the abrupt change of routine during the COVID-19 pandemic. As Di Fabio & Tsuda ([6]; pp. 4) said: within the individual level, “harmony refers to the person and the process of harmonizing various parts of the body, mind-heart, and different purposes in life in a well-functioning organic whole”. The imbalance that has arisen between online and offline life can thus be seen as a process of disharmonization. 

The pandemic scenario and the consequential lack of opportunities for offline socialization have led to greater use of ICTs. In some cases, this has led researchers to worry about the emergence of problematic use of and addiction to these technologies, and therefore to a situation identifiable as dis-equilibrium [9]. People who are “technology addicted” are defined as those who have difficulty reducing ICT use even when it interferes with work, study, psychophysical well-being, or social function [10]. The dysfunctional use of ICTs has attracted increasing attention in popular media and among researchers studying behavioral addiction [11].

In general, a “behavioral addiction” fully falls into the category of addictions; in this case, the individual is not addicted to a substance but rather to outcomes or sensations experienced by implementing certain behaviors [12]. Young [13] argued that “technology addiction” is difficult to identify as the use of ICTs has penetrated our daily way of life, such that its growing and predominant use is not always synonymous with addiction. What determines the boundary between habit and addiction is the behavioral model that characterizes dysfunctional use of ICTs, which shares similarities with the behavioral model that characterizes substance addiction [14]; both types of addictions present withdrawal symptoms, such as the repeated inability to reduce the frequency of use or stop it altogether, and an inevitable impairment of daily life [15,16]. Similar to substance addiction, addiction to ICTs also involves a degenerative behavioral and emotional path that hinders one’s ability to achieve a balance between work and family and that can create a sense of emotional exhaustion. Attention must be paid to the label of “addiction” since, despite the evidence reported so far which suggests that the internet and new technologies can be considered a source of addiction, there is other works that has questioned this interpretation [17]. 

However, it has been shown that dependence on ICTs, in particular social media, is significantly correlated to the harmonic balance between work and family [18]. Therefore, introducing the concept of “Digital Life Balance” (DLB) appears necessary given the current increase in WFH hours [19] and ICT use [20,21] due to the COVID-19 pandemic, as well as the availability and increasing pervasiveness of ICTs [22]. Starting from the concept of Work-Life Balance often used in organizational psychology [23], DLB is here defined as the perceived balance between online and offline life. The concept of DLB aims to reconcile and enhance two important lines of research. On the one hand, DLB may be used to investigate problematic or addictive technology use that is often captured through measures of screen time (Seaward, 2020) or dedicated assessment tools such as the Bergen Social Media Addiction Scale (BSMAS) [24], both of which may be less effective due to the aforementioned increase in ICTs [25]. On the other hand, DLB aims to capture the other possible dimensions of ICT use (i.e., adaptive, harmonic, and profitable [26]). 

## 2. Aim and Hypotheses Development

### 2.1. Aim of the Study 

The aim of this study was to first develop a scale that could capture people’s harmonic (i.e., balanced) and disharmonic (i.e., unbalanced) use of ICTs and then to validate it internally and externally. 

### 2.2. Hypotheses Development

To accomplish these goals, we conducted two studies: one aimed at assessing the internal validity of the scale (i.e., factorial structure, invariance, and reliability), and another focusing on the external validity. Based on the literature, we expected that DLB scores would be related to two main clusters of measures: well-being and addiction related. More specifically, since non-harmonious and dysfunctional ICT use results in a reduction in or a total loss of well-being [27,28], we expected that lower DLB scores would be related to lower levels of affective hedonic (i.e., PANAS; [29]), cognitive hedonic (i.e., SWL; [30]), and eudaimonic (i.e., Flourishing; [31]) well-being. 

Consequently, the first three hypotheses regarding the external validity of the DLB Scale were the following:

**H1.**  *DLB scores have a positive relationship with PANAS positive scores and a negative relationship with PANAS negative scores.*

**H2.**  *DLB scores have a positive relationship with scores on the Satisfaction with Life Scale.*

**H3.**  *DLB scores have a positive relationship with scores on the Flourishing Scale.*

Moreover, since low levels of balance appear to be linked to dysfunctional use of ICTs [18,32,33], we expected high levels of DLB to be negatively associated with scores on scales measuring online addictions [34,35], whether it be an addiction to social media, smartphones, the internet in general, or gaming activity. Therefore, we formulated four hypotheses regarding the external validity:

**H4.**  *DLB scores have a negative relationship with scores on the BSMAS.*

**H5.**  *DLB scores have a negative relationship with scores on the Gaming Addiction Scale.*

**H6.**  *DLB scores have a negative relationship with scores on the Internet Addiction Scale.*

**H7.**  *DLB scores have a negative relationship with scores on the SABAS.*

## 3. Methods and Procedure

### 3.1. Measures 

#### 3.1.1. Digital Life Balance Scale—DLB Scale

The scale consists of four items measured on a 7-point Likert scale ranging from 1 (strongly disagree) to 7 (strongly agree). This set of items was developed starting from the Work-Life Balance Scale by Brough et al. [36]. 

This scale was selected among the many existing work-life balance scales because of its brevity of administration (e.g., the 14 items of the Work Interference with Personal Life (WIPL) Scale [37] or the 11 items of the Women’s Work-Life Balance Scale [38]); the number of times it has been cited (318 citations on Google Scholar in November 2022), which indicates a wide adoption of this scale by the scientific community; and how the items were formulated, which was done it a way that was particularly suitable for the adaptation that the authors had in mind. The original items were restructured replacing the wording “work and non-work” and “working and non-working” with “online and offline”. Like the Brough et al. [36] scale, the DLB Scale scoring range varies between a minimum of 4 and a maximum of 28 and is calculated through the sum of the four items after reversing item 2. The scale items are presented in Table 1. 

#### 3.1.2. Satisfaction with Life Scale (SWL—[39])

We used the validated Italian version of this scale [40] to measure people’s sense of satisfaction with their lives, which is a very important factor in determining their subjective well-being. It is a one-dimensional scale that involves 5 items (e.g., ”I am satisfied with my life”, ”The conditions of my life are excellent”) that are rated using a 7-point Likert scale (1 = strongly disagree; 7 = strongly agree). The analysis of the scale’s reliability showed good internal consistency (α = 0.74) [41]; in our study, the α was 0.89. The scoring range varies between a minimum of 5 and a maximum of 35, where high scores correspond to greater life satisfaction.

#### 3.1.3. Flourishing Scale [42]

This scale has been validated in Italian by Di Fabio [43]. It aims to measure the construct of well-being, especially in the psychological domain, with questions related to self-esteem, optimism, relationships, and purposes. It is a one-dimensional scale that includes 8 items (e.g., “I lead a full and meaningful life”, “People respect me”) that are rated using a 7-point Likert scale (1 = strongly disagree; 7 = strongly agree). Internal consistency coefficients for the scale have ranged from 0.82 to 0.82 [44]; in our study, the α was 0.89. The scoring range varies between a minimum of 8 and a maximum of 56, where high scores correspond to greater well-being. 

#### 3.1.4. PANAS [45]

This scale has been translated and validated in Italian by Terraciano and colleagues [46]. The Positive and Negative Affect Schedule is a scale designed to gauge a person’s emotional or negative activation. Positive and negative activation are emotional components of psychological and subjective well-being. It has two subscales: Positive Affect and Negative Affect. It is composed of 20 items, with 10 items measuring positive affect (e.g., excited, inspired) and 10 items measuring negative affect (e.g., upset, afraid). Items are rated using a 5-point Likert scale (1 = strongly disagree; 5 = strongly agree) [47]. Reliability and validity reported by Watson et al. [45] were good; for the Positive Affect Scale, the Cronbach’s alpha coefficient was 0.86 to 0.90 (α = 0.83 in our study); for the Negative Affect Scale, the coefficient was 0.84 to 0.87 [45] (α = 0.89 in our study). The scoring range varies between a minimum of 10 and a maximum of 50 for each subscale, where high scores correspond to greater emotional activation. 

#### 3.1.5. Bergen Social Media Addiction Scale (BSMAS—[48])

This scale, translated and validated in Italian by Monacis et al. [49], is useful for measuring a person’s degree of dependence on social networks. It is made up of 6 items (e.g., “I don’t get tired of playing video games ”,”I lose track of time when I play”) that are rated using a 5-point Likert scale (1 = strongly disagree; 5 = strongly agree). The internal consistency coefficient for the scale is α = 0.86 [50] (α = 0.83 in our study) and the scoring range varies between a minimum of 6 and a maximum of 30, where high scores correspond to greater addiction. 

#### 3.1.6. Smartphone Application Based Addiction Scale (SABAS [51])

The Smartphone Application Based Addiction Scale was produced to study a person’s degree of dependence on smartphones and applications. For our studies, we used the version validated in Italian by Soraci and colleagues [52]. The SABAS consists of 6 items (e.g., “My smartphone is the most important thing in my life “,” I feel the need to spend more and more time using my smartphone”) that are rated on a 6-point Likert scale (1 = strongly disagree; 6 = strongly agree). The internal reliability of the scale is good, with a Cronbach’s alpha coefficient of 0.81 [51] (α = 0.86 in our study), and the scoring range varies between a minimum of 6 and a maximum of 36, where high scores correspond to greater addiction.

#### 3.1.7. Internet Addiction Scale [53]

The Internet Addiction Scale measures the presence and severity of internet addiction. The scale consists of 6 items (e.g., “I feel anxious when I don’t have internet access”,”I spend more time on the internet than planned”) that are rated using a 5-point Likert scale (1 = strongly disagree; 5 = strongly agree). Italian items have been preliminarily validated by Guazzini et al. [54], and all the items load on to only one factor. Cronbach’s alpha coefficient for the Internet addiction scale was 0.83 [54] (α = 0.80 in our study) and the scoring range varies between a minimum of 6 and a maximum of 30, where high scores correspond to greater addiction.

#### 3.1.8. Gaming Addiction Scale [53]

It measures the degree of dependence on video games and consists of 8 items (e.g.,”I lose track of time when I play”, “I postpone bedtime to play”) that are rated using a 5-point Likert scale (1 = strongly disagree; 5 = strongly agree). Italian items have been preliminarily validated by Guazzini et al., [54], and all the items load on to only one factor. The Cronbach’s alpha coefficient for the Game Addiction Scale was 0.90 [54] (the same reliability was also obtained in our study), and the scoring range varies between a minimum of 8 and a maximum of 40, where high scores correspond to greater addiction.

### 3.2. Sample and Sampling

Before proceeding with recruitment for both Study 1 and Study 2, we defined an adequate sample size for each of them. For Study 1, at least a 10:1 ratio between participants and items was recommended [55] and a sample size higher than 200 was identified as being “fair” enough to run confirmatory factor analysis [56,57]. Since we were able to gather data from 1473 participants, we deemed the sample size for Study 1 to be adequate. To define the appropriate sample size for Study 2, we performed a power analysis using G*Power [58,59]. Since the authors planned to use Pearson’s correlations to investigate the relationship between DLB scores and external validity, a power analysis was computed for this type of analysis. The power analysis showed that a sample size of 782 would be required to achieve a statistical power of 0.80 while being able to capture even a small effect size (r = 0.10) and assuming a significance level of 0.05. Additionally, we accounted for the required sample size for achieving a stable measurement-error-free correlation. In our case (i.e., population correlation q = 0.10; composite score reliability derived from other works ω = 0.80), a stable measurement-error-free correlation would be met at 380 [60]. Since the number of participants recruited for Study 2 was 953, we deemed our sample size to be adequate for our research purposes. The recruitment strategy for both studies was the same. Participation was promoted through posts and messages on social media platforms, like Facebook and Instagram, as well as by directly asking people to participate by scanning a QR-code which led to the online data collection form. Data were collected following the Italian law’s privacy requirements (Law Decree DL-101/2018) and EU regulations (2016/679). In the first study, 1473 people (64.9% female), with an average age of 29.72 (s.d. = 12.24; age range = 14–82), participated and completed the survey. 953 people (64.3% females) participated in Study 2 (average age = 29.50; s.d. = 11.57; age range = 15–80).

### 3.3. Data Analysis

In the first study, we performed confirmatory factor analysis to assess DLB dimensionality using AMOS software. Maximum likelihood estimation (MLE) was used to estimate the model’s parameters. To evaluate the models, several goodness-of-fit indices were used: the Chi square to degree of freedom ratio (χ^2^/df; [61]), the Tucker–Lewis index (TLI; [62]), the standardized root mean square residual (SRMR; [63]), the root mean square error of approximation (RMSEA; [64]), and the comparative fit index (CFI; [65]). The thresholds used to deem models as adequate were the following: a TLI value higher than 0.95, a CFI value close to 0.95 (0.90 to 0.95 for a good fit), a SRMR value less than 0.08, and a RMSEA less than 0.06 (0.06 to 0.08 for a good fit; [66]). DLB sex invariance was assessed through multigroup confirmatory factor analysis. In the second study, we relied on Pearson’s bivariate and partial correlations to investigate relationship between DLB scores and external validity measures. 

## 4. Results

### 4.1. Study 1

#### 4.1.1. Items Descriptive Statistics

As a first step, we produced the descriptive statistics for all the items involved in our data collection (Table 1). 

#### 4.1.2. Confirmatory Factor Analysis (CFA)

**Figure 1:**
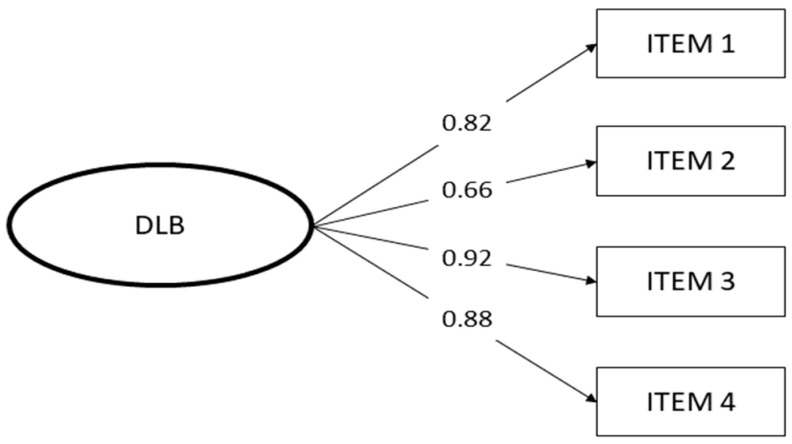
Results of confirmatory factor analysis of the DLB Scale.

Since the Digital Life Balance Scale was developed using the Work-Life Balance Scale, we assumed that the two scales would share the same dimensionality (i.e., factorial structure). For this reason, CFA was performed to test the one-factor structure. Maximum likelihood estimation (MLE) was used to estimate the model’s parameters. The CFA showed an optimal fit for the DLB one-factor model (χ^2^/df = 4.38; *p* = 0.012; TLI = 0.99; CFI = 0.99; RMSEA = 0.0048; SRMR = 0.0097). Moreover, all factor loadings were statistically significant and higher than the conventionally acceptable threshold of >0.50 (Figure 1).

#### 4.1.3. Sex Invariance

We tested DLB score invariance using multigroup confirmatory factor analysis considering sex. Three levels of invariance were tested (i.e., configural, metric, and scalar) by relying on changes in RMSEA and CFI since the Chi-square test is sensitive to sample size [67,68]. Changes in model fit indices should be less than 0.002 for the CFI [69] and less than 0.010 for the RMSEA [67]. For the sex variable, both the difference between the configural and metric models (ΔCFI  =  0.001; ΔRMSEA  =  0.01) and the difference between the metric and scalar models (ΔCFI  <  0.001; ΔRMSEA  = 0 .008) were not significant. Overall, the Italian version of the DLB Scale appeared to be invariant with respect to the sex dimension.

#### 4.1.4. Reliability Assessment

The reliability analysis of the DLB one-factor model was carried out by calculating McDonald’s omega. This was done based on the consensus in the psychometric literature that Cronbach’s alpha is rarely appropriate [70,71,72]. The DLB Scale showed an optimal reliability (ω = 0.89). 

### 4.2. Study 2

#### External Validity

The external validity of the DLB Scale was assessed using Pearson’s r correlations as envisaged in our hypotheses. Before proceeding with correlational analysis, we assessed the normality (asymmetry and kurtosis values), homoscedasticity, and linearity of each variable and produced descriptive statistics (Table 2).

Since all the metric variables were normally distributed, we performed Pearson’s correlation tests as envisaged. To test the consistency of the correlations, we also carried out partial correlation tests controlling for participants’ age and sex (Table 3). As expected, DLB was negatively correlated with addiction-related measures and positively correlated with well-being measures, with the obvious exception of PANAS negative activations (negative relationship). According to the literature on interpretation rules for Pearson’s correlations in social sciences [73], the DLB scores showed relatively strong relationships with external validity measures, with the relationship between DLB and gaming addiction being the only one with a “typical” effect size. Subsequent bivariate correlations did not differ greatly from the partial correlations, thus suggesting that participants’ age and sex had, at most, a minimal effect on the relationship. 

## 5. Discussion

### 5.1. Discussion

Since the start of the Digital Revolution, to the recent COVID-19 pandemic, people across the world, regardless of field, are becoming increasingly reliant on online activities. Even in light of the pandemic emergency situation which forced millions of people to use the internet for both work and private life (e.g., during the lockdown and stay-at-home periods), both the number of people connected to the internet (+4%) and those registered on social platforms (+10%) increased in 2022 compared with previous years. [74]. As highlighted by the results of the Digital Global Overview report [74], people across the world have increased their activities on the internet and social networks both for work and fun and as a method of maintaining social relationships [75]. Users reported using platforms such as Youtube, Facebook, and Whatsapp for about 20 hours per month [74] and that they actually prefer working from home, either completely or partially [76]. However, according to previous studies [77,78,79,80,81,82,83,84], massive use of these technologies can lead to the development of problematic use and addiction that creates psychological, social, school, and work difficulties in a person’s life [77]. In the same way as substance addiction, addiction to ICTs also involves a degenerative behavioural and emotional path that hinders a person’s ability to achieve a balance between work and family and that can create a sense of emotional exhaustion [18]. The imbalance between online and offline life may reflect a dysfunctional use of ICTs and can be seen as a process of disharmonization. However, people can also use ICTs in a functional way. Indeed, a positive relationship between internet use and well-being was previously reported in the literature [85,86,87,88,89]. Furthermore, functional use of ICTs can lead to a harmonic balance between digital life and real life, which is related to well-being and can positively influence people and promote life satisfaction and psychological well-being [90]. Therefore, it was necessary to create a scale that could define an individual’s perceived balance between online and offline life.

In Study 1, we tested the internal validity of the DLB Scale using a confirmatory factor analysis (CFA). In line with the factorial structure of the Work-Life Balance Scale [36], our results appears to support the one-factor structure of the. The DLB Scale also showed optimal reliability (ω = 0.89) and demonstrated sex invariance.

In Study 2, we assessed the external validity of the Digital Life Balance Scale using existing well-being and ITC addiction scales. As expected, the results showed a positive relationship between DLB scores and levels of both hedonic and eudaimonic well-being [29,30,31]. Specifically, low DLB scores were related to low scores on the Satisfaction with Life Scale (r = 0.32), Flourishing Scale (r = 0.38) and PANAS positive scale (r = 0.28). In contrast, PANAS negative scores showed a negative correlation with DLB (r = −0.31), as expected from the literature [29]. In this study, we also tested the relationship between the DLB Scale and scales assessing the dysfunctional use of ICTs. In line with the literature [34,35], DLB scores appeared to be negatively associated with ICT addiction levels measured with the BSMAS scale (r = −0.40), the Gaming Addiction Scale (r = −0.23), the Internet Addiction Scale (r = −0.42), and the SABAS scale (r = −0.38). 

### 5.2. Limitation and Future Studies

Obviously, our external validation results are correlational and no causation among variables can be inferred. Moreover, our results are based on a biased sample due to non-random sampling and self-selection bias. Therefore, the generalizability of our results may be limited. Future research should deal with these limitations and investigate how DLB scores relate to the number of WFH hours. Moreover, since underlying psychopathology appears to be a risk factor for problematic or addictive use of technology, the relationships between digital life balance and obsessive-compulsive disorder [91,92,93,94], depression [94,95,96], anxiety disorders [97], and attention deficit hyperactivity disorder (ADHD) [97,98] should be explored in the near future. 

## 6. Conclusions

In conclusion, despite the aforementioned limitations, the DLB Scale appears to be a reliable and valid instrument to investigate people’s harmonic and disharmonic use of ICTs.

## Figures and Tables

**Table 1:** Descriptive statistics of the item pool used to build the Digital Life Balance Scale.

N°	Item	Min	Max	Mean	s.d.
1	ENG: I currently have a good balance between the time I spend online and the time I have available for offline activities. IT: Attualmente ho un buon equilibrio fra il tempo che spendo online e quello disponibile per le attività offline	1	7	4.94	1.62
2	ENG: I have difficulty balancing my online and offline activities. [R] IT: Ho problemi a bilanciare le mie attività online e quelle offline. [R]	1	7	5.07	1.74
3	ENG: I feel that the balance between my online and offline activities is currently about right. IT: Ritengo che l’equilibrio fra le mie attività online e offline sia adeguato	1	7	5.03	1.71
4	ENG: Overall, I believe that my online and offline life are balanced. IT: Tutto sommato ritengo che la mia vita online e offline sia bilanciata	1	7	5.16	1.68

Note: N = 1473; s.d. = standard deviation; ENG = English version of the items (not yet validated); IT = Italian version of the items (validated in this paper); [R] = reverse item.

**Table 2:** Descriptive statistics of the variables collected in study 2.

Variables	Min	Max	Mean	s.d.	Asym.	Kurt.
Digital Life Balance Scale	4	28	19.33	5.76	−0.40	−0.69
Satisfaction with Life Scale	5	35	22.85	6.56	−0.48	−0.22
Flourishing Scale	11	56	42.70	7.95	−0.92	0.98
PANAS-Positive	14	50	37.96	5.83	−0.24	0.26
PANAS-Negative	10	50	24.16	8.11	0.50	−0.14
BSMAS	6	30	13.63	5.58	0.63	−0.29
SABAS	6	36	11.36	5.64	0.98	0.97
Internet Addiction Scale	6	30	12.55	4.84	0.75	0.11
Gaming Addiction Scale	8	40	15.14	7.40	0.90	−0.19

Note: N = 953; s.d. = standard deviation; Asym. = asymmetry; Kurt. = kurtosis; BSMAS = Bergen Social Media Addiction Scale; SABAS = Smartphone Application Based Addiction Scale.

**Table 3:** Pearson’s correlation analysis for external validity assessment.

Variables	Digital Life Balance Scale	Digital Life Balance Scale (Controlled for Age and Sex)
Satisfaction with Life Scale	0.32 ***	0.31 ***
Flourishing Scale	0.38 ***	0.37 ***
PANAS—Positive	0.28 ***	0.26 ***
PANAS—Negative	−0.31 ***	−0.29 ***
BSMAS	−0.40 ***	−0.38 ***
SABAS	−0.38 ***	−0.36 ***
Internet Addiction Scale	−0.42 ***	−0.40 ***
Gaming Addiction Scale	−0.23 ***	−0.22 ***

N = 953; *** = *p* < 0.001; BSMAS = Bergen Social Media Addiction Scale; SABAS = Smartphone Application Based Addiction Scale.

## Data Availability

The data presented in this study are available on request from the corresponding author.
